# *Vibrio vulnificus* and *Vibrio parahaemolyticus* in Oysters under Low Tidal Range Conditions: Is Seawater Analysis Useful for Risk Assessment?

**DOI:** 10.3390/foods11244065

**Published:** 2022-12-16

**Authors:** Corinne Audemard, Tal Ben-Horin, Howard I. Kator, Kimberly S. Reece

**Affiliations:** 1 Virginia Institute of Marine Science, William & Mary, P.O. Box 1346, Gloucester Point, VA 23062, USA; 2 College of Veterinary Medicine, North Carolina State University, Morehead City, NC 28557, USA

**Keywords:** *Crassostrea virginica*, *Vibrio vulnificus*, *Vibrio parahaemolyticus*, model, aquaculture, seafood safety, ecology, tidal range

## Abstract

Human-pathogenic *Vibrio* bacteria are acquired by oysters through filtering seawater, however, the relationships between levels of these bacteria in measured in oysters and overlying waters are inconsistent across regions. The reasons for these discrepancies are unclear hindering our ability to assess if -or when- seawater samples can be used as a proxy for oysters to assess risk. We investigated whether concentrations of total and human pathogenic *Vibrio vulnificus* (*vvhA* and *pilF* genes) and *Vibrio parahaemolyticus* (*tlh*, *tdh* and *trh* genes) measured in seawater reflect concentrations of these bacteria in oysters (*Crassostrea virginica)* cultured within the US lower Chesapeake Bay region. We measured *Vibrio* spp. concentrations using an MPN-qPCR approach and analyzed the data using structural equation modeling (SEM). We found seawater concentrations of these bacteria to predictably respond to temperature and salinity over chlorophyll *a*, pheophytin or turbidity. We also inferred from the SEM results that *Vibrio* concentrations in seawater strongly predict their respective concentrations in oysters. We hypothesize that such seawater-oyster coupling can be observed in regions of low tidal range. Due to the ease of sampling and processing of seawater samples compared to oyster samples, we suggest that under low tidal range conditions, seawater samples can foster increased spatial and temporal coverage and complement data associated with oyster samples.

## 1. Introduction

Oysters acquire human-pathogenic *Vibrio* species naturally occurring in seawater through their filter-feeding activity and when consumed raw, are a vector for these pathogens [1]. The two main species of concern with regard to oyster consumption, *Vibrio vulnificus* and *V. parahaemolyticus*, are associated with illnesses ranging in severity from mild gastroenteritis to septicemia that can occasionally become fatal [1]. The number of illness cases associated with *V. vulnificus* is low (~100 cases/year), however, due to a 30% fatality rate, *V. vulnificus* is the leading cause of seafood-borne mortality in the US [1,2,3,4,5]. In contrast, *V. parahaemolyticus* is associated with seafood-borne gastroenteritis, but the caseload for this species is high with an estimated 45,000 cases per year [1]. In situ dynamics of these bacteria have been shown to be influenced by seawater temperature and salinity, however, previous studies also highlighted the limitations of these two environmental factors in fully explaining the dynamics of these bacteria [6].

While one could hypothesize that concentrations of *Vibrio* bacteria in oysters are influenced by levels observed in overlying water, the existence of an association—or the degree to which levels of these bacteria in oysters are associated with concentrations measured in water—requires further investigation. The relationships and potential coupling between the concentrations of *Vibrio* spp. in seawater and in oysters has rarely been explored and led to contrasting results depending on the region [7,8,9]. Indeed, some studies conducted along the Gulf and East coasts of the US observed an association between concentrations of these bacteria in seawater and oyster [7,9], while a study conducted on the Pacific Northwest coast revealed an absence of association [8]. Gaining a better understanding of the factors driving these observations can shed light on the potential connectivity and dynamics of these bacteria in different ecological niches or habitats but such studies also have direct applications to risk management. More specifically, these studies can help determine the value of seawater analysis for *Vibrio* spp. levels in the context of risk assessment. Indeed, a well-defined association between concentrations measured in oysters and in overlying seawater would enable the use of predictive models based on *Vibrio* spp. levels in seawater samples to assess human-health risk associated with raw oysters. Water-based predictive models such as those focusing on the Chesapeake Bay region [10,11,12], generally rely on extensive datasets by taking advantage of the relative ease of collection and processing of water samples compared to oyster samples. These models are inherently relevant for assessing risks associated with water exposure, however, in the absence of a well-defined relationship between seawater and oyster *Vibrio* concentrations, the relevance of water-based models to risk management associated with raw oyster consumption is limited. In addition to the relevance to predictive models, a demonstrated association between the two matrices would legitimize the monitoring of water in addition to oyster samples, making it feasible to decrease processing time and to enhance sampling frequency and spatial coverage to ultimately improve risk assessment [7].

Our aim here is to understand the interaction between seawater and oyster tissue-associated *Vibrio* spp. under the abiotic and biotic conditions observed within the lower Chesapeake Bay region. In doing so, our objective is also to contribute to the body of work focusing on the ecology of these bacteria to identify the factors driving their temporal and geographical distribution [6]. Pathogenic *V. parahaemolyticus* strains were measured through the detection of the commonly used thermostable-direct hemolysin (*tdh*) and thermostable-direct related hemolysin (*trh*) genes [13]. For pathogenic *V. vulnificus* strains, our gene target was the pilus-type assembly IV gene (*pilF*) shown to have comparable sensitivities and specificities as other *V. vulnificus* pathogenicity markers [14]. We used structural equation modeling (SEM), a multiequational framework designed for testing multivariate hypotheses, to account for the roles of abiotic and biotic factors on *Vibrio* spp. in seawater and associated oyster tissue. This approach is used widely in ecology to address the complex relationships between broad environmental variables [15] such as climate [16,17,18] and eutrophication [19] and measurable outcomes such as species diversity, environmental productivity, food web complexity, species abundances, and in our case, microbial concentrations in the environment and seafood. We began with a conceptual model of expected multivariate relationships between seawater and oyster tissue-associated *Vibrio* and their environment, and then adapted this model to the collected data describing the abiotic (temperature and salinity) and biotic (primary productivity, quantified as chlorophyll *a*, pheophytin and/or turbidity) environment. This approach allowed us to address specific questions of the relative importance of abiotic and biotic factors driving total and human-pathogenic *V. parahaemolyticus* and *V. vulnificus* concentrations in oysters and seawater. Additional analyses were used to examine associations between total and human-pathogenic *V. parahaemolyticus* and *V. vulnificus* concentrations within oysters. SEM results highlighted a positive association between levels of each *Vibrio* target measured in seawater and oysters at our studied sites. Within the oyster habitat, we also observed a strong association between levels of total and pathogenic *V. vulnificus* strains but an absence of association in the case of *V. parahaemolyticus*.

## 2. Materials and methods

### 2.1. Study Sites

The study was conducted within the mid-Atlantic region of the US, at three sites located within the lower Chesapeake Bay and one site located in a seaside bay on the Eastern Shore (Delmarva Peninsula), Virginia. These sites encompassed a mesohaline site (or low salinity site) located on the western shore and two polyhaline (or moderate salinity) sites; one located in the York River on the lower western side of the Bay (Site 1) and one located in a creek on the lower eastern side of the Bay (Site 2). The fourth site located on the seaside was exposed to euhaline (or high salinity) water. The location of these sites is not disclosed following an agreement with the oyster-growers who contributed to the study.

Oysters were reared using common aquaculture practices such as off-bottom cages (low salinity site and moderate salinity Site 2) or bags on racks (moderate salinity Site 1 and high salinity site). The oysters grown at the moderate salinity Site 1 and high salinity site were exposed to air at low tide for <2 h during spring tides. Collection of oysters was not conducted at a particular time with regard to the tide. Water depth at each site ranged from 1.2 to 1.8 m. Oyster and surface water samples were collected from April through November in 2012 (Year 1), and in 2014 (Year 2), however, study sites and sampling regime varied between years. In Year 1, collection was conducted from the three Chesapeake Bay sites (low, and both moderate salinity sites) on a monthly basis, while in Year 2, samples were collected from 2 of the Bay sites (low and moderate salinity Site 1) and from the high salinity site on a biweekly basis.

### 2.2. Sample Collection

At each site and time point, samples consisted of four replicate samples of ten oysters each and one surface water sample (100 mL). Overall, 284 replicate oyster samples and 71 water samples were collected over the course of this study. Oysters and water samples were kept chilled in insulated coolers and separated from direct contact with ice during transport. Water temperature and salinity were measured from each site during both years of the study. Water temperatures were obtained from in situ temperature loggers (HOBO, Onset Inc., Bourne, MA, USA), from sondes deployed at some of the sites by the NOAA National Estuarine Research Reserve System [20], or from hand held thermometer records. Salinity was measured upon collection of the samples using a calibrated refractometer (Cole-Parmer, Vernon Hills, IL, USA).

In addition, in 2014, chlorophyll *a*, pheophytin and turbidity were measured on water samples collected at each sampling time point. Chlorophyll *a* and pheophytin concentrations were measured following the method 445.0 described by US Environmental Protection Agency (US EPA) [21]. Briefly, this method involves the filtration of a water sample on a glass fiber filter (Sigma-Aldrich, St. Louis, MO, USA), the extraction of the pigments from the filter and fluorescence measurements on a fluorometer (Turner Designs, San Jose, CA, USA). Turbidity was determined by nephelometry following method 180.1 as described by US EPA [22]. The approach relies on intensity measures of the light scattered by a sample in comparison to a standard reference suspension.

### 2.3. Sample Processing

Levels of total and pathogenic *V. vulnificus*, and levels of total and pathogenic *V. parahaemolyticus* in oyster and water samples were determined using a most-probable number (MPN) approach followed by quantitative PCR (qPCR). For each replicate oyster sample, a tissue homogenate was prepared by pooling the tissues and liquor of ten oysters. Homogenates and decimal dilutions (1 × 10^−1^ to 1 × 10^−7^ g) thereof were inoculated into an alkaline peptone water (APW) MPN series as described in the FDA Bacteriological Analytical Manual [23]. For each site, a water sample was analyzed and decimal dilutions (1 mL to 1 × 10^−6^ mL) were inoculated as described above for the oyster homogenates. Following incubation at 35 °C for 18–24 h, a 1 mL volume was removed from the top cm of each APW enrichment tube showing turbidity and boiled for 10 min to lyse cells [24]. This lysate was subsequently used as the template DNA sample in each of the qPCR assays described below. Results of the qPCRs were used to assess the MPN density values using approved MPN tables [23]. Based on the range of dilutions analyzed and the number of tubes per dilutions (here 3 tubes), the detection limit given using the MPN tables was 3.0 MPN/g for the oysters and 0.3 MPN/mL for the water samples.

Detection of total *V. vulnificus* in APW enrichment lysates was performed by targeting the hemolysin/cytolysin gene (*vvhA*) using the TaqMan^®^ assay designed by Campbell and Wright [25] (Table S1). Conditions were as described in Audemard et al. [26] except that TaqMan^®^ Fast Advanced Master Mix (Life Technologies, Grand Island, NY, USA) was used. For the detection of potentially pathogenic *V. vulnificus* strains, we use the assay described by Baker-Austin et al. [27] targeting the gene coding for the protein required for pilus-type assembly IV (*pilF*) (Table S1). Polymorphism within the *pilF* gene has been used to distinguished pathogenic and non-pathogenic strains independent of the biotype [28]. A recent study by Dickerson et al. [14] showed that this marker is as efficient in detecting pathogenic strains as markers based on 16S rRNA polymorphism [29,30,31], or as markers based on a virulence-coded gene [32,33,34]. The *pilF* assay developed by Baker-Austin et al. [22] was also chosen because it was developed for application to oyster and water samples (with and without enrichment) and did not require the isolation of strains. This qPCR assay was run on the lysates following the published conditions except for the template and reaction volumes which were reduced to 1 μL and 10 μL, respectively.

We used a modification of the qPCR assay described by Nordstrom et al. [13] to detect the presence of *V. parahaemolyticus* and pathogenic strains in each lysate (Table S1). We used a multiplex assay to detect the presence of the thermolabile hemolysin gene (*tlh*), a marker found in all *V. parahaemolyticus* isolates, and the *tdh* gene encoding thermostable direct hemolysin, a virulence marker associated with a majority of clinical cases [35,36]. Conditions were as described in Audemard et al. [26] although we used the TaqMan^®^ Fast Advanced Master Mix (Life Technologies, Grand Island, NY, USA). We used a separate qPCR assay to detect the presence of the *trh* gene encoding *tdh*-related hemolysin, another virulence marker associated with a majority of clinical isolates [37,38]. Detection of *trh* was performed using the primers, probe, and cycling conditions described by Nordstrom et al. [13] with the following master mix final concentrations: bovine serum albumin at 0.4 µg/µL, 1× TaqMan^®^ Fast Advanced Master Mix (Life Technologies, Grand Island, NY, USA), 0.3 µM of primers and 0.15 µM of the probe, 1 µL of the template and a final reaction volume of 10 µL.

All oyster (*n* = 284) and water samples (*n* = 71) collected during this study were analyzed for *vvhA*, *tlh*, *tdh* and *trh*. All oyster (*n* = 192) and water samples (*n* = 48) collected in Year 2 were analyzed for *pilF*, however, only a subset of the oyster (30 out of 92), and water samples (9 out of 23) samples were analyzed for this marker in Year 1.

### 2.4. Data Analyses

Data were graphically presented and figures prepared using R [39]. For samples associated with *Vibrio* concentrations below the detection limit of the MPN qPCR assays, either <3 MPN/g for oyster samples or <0.3 MPN/mL for the water samples, a value of 1.5 MPN/g or 0.15 MPN/mL, respectively was given for graphical presentation of the data.

The relationships between concentrations of *vvhA*+ and *pilF*+ *V. vulnificus* strains and between concentrations of *tlh*+ and *tdh*+ or *trh*+ *V. parahaemolyticus* strains in oysters were visually assessed and tested for linear associations by Pearson correlations. This allowed us to test whether measured *Vibrio* concentrations in oysters were associated with elevated concentrations of pathogenic *Vibrios*.

**Figure 1:**
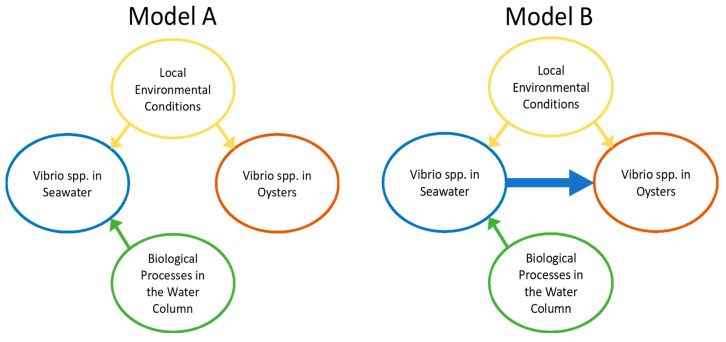
Conceptual model generalizing the expected relationships between environmental factors (e.g., temperature and salinity) and biological processes (e.g., abundance of host organisms and carbon available for decomposition) that drive *Vibrio* spp. abundance both in the water column and in oysters. Arrows indicate which factors are influencing *Vibrio* levels in seawater and oysters in each model. Two competing models are represented; Model A (**left**) represents a null expectation that *Vibrio* spp. in seawater and oysters are driven only by local environmental conditions. Model B (**right**) builds on this expectation by including an additional path representing the case where *Vibrio* spp. concentrations in oysters respond to concentrations of *Vibrio* spp. in seawater.

Beyond these linear relationships, we sought here to understand (1) how various abiotic and biotic factors were related to concentrations of *Vibrio* spp. in seawater, and (2) how these environmental factors and *Vibrio* spp. levels in seawater were related to *Vibrio* spp. abundance in oysters. To address these questions, we began with a conceptual model (Figure 1) based on established work [6], generalizing the expected relationships between environmental conditions (e.g., temperature and salinity) and biological processes (e.g., abundance of host organisms and carbon available for decomposition) that drive *Vibrio* spp. abundance both in the water column and in oysters. We used this general model to represent two competing hypotheses: Model A represents a null expectation that *Vibrio* spp. in seawater and oysters are driven only by environmental conditions. Model B builds on this expectation but includes an additional path representing the case where *Vibrio* spp. concentrations in oysters respond to concentrations of *Vibrio* spp. in seawater.

We then adapted this general model to the particulars of our collected data using structural equation modeling (SEM), allowing us to relate our observed biotic (primary productivity measured as chlorophyll *a*, pheophytin, and/or turbidity) and abiotic (temperature and salinity) environmental variables in our model construct. In our preliminary analyses we found our measured values of chlorophyll *a*, pheophytin, and turbidity to be correlated, which was expected. We therefore only considered these variables in models individually, and interpreted results based on our ecological understanding of these variables and the potential bias from their use as proxies for primary productivity. In addition, based on previous work [40], we considered a linear relationship between salinity and seawater *V. vulnificus* concentrations (*vvhA*+ and *pilF*+) but a convex relation between salinity and *V. parahaemolyticus* (*tlh*+, *tdh*+, and *trh*+). The latter models considering relationships between salinity and *V. parahaemolyticus* therefore had two salinity terms (one linear and one squared term). The parameters describing these environmental relationships were derived from the fitted models.

We derived the SEM equations and implemented these in the statistical software R (version 4.1.3, R Core Team, Vienna, Austria) [39] using the Lavaan package [41]. The adequacy of model fit was evaluated using the model chi-square and associated *p* values, as well as examination of deviations between observed and expected covariances. We examined other fit indices such as the Akaike’s Information Criterion, AIC [42] in preliminary models and found these to be consistent with the chi-square test results. Individual path coefficients were also evaluated using z-tests. Our analysis was guided by the two models represented in Figure 1, with Model A representing null expectations of *Vibrio* spp. concentration in oysters being driven entirely by abiotic and biotic environmental conditions. Model B included the additional paths between seawater *Vibrio* spp. and *Vibrio* spp. in oysters. Ultimately, results and interpretations presented in this report were based on the models judged to be the best representation of the data using model comparisons and objective measures of model fit.

## 3. Results

### 3.1. Environmental Parameters

**Figure 2:**
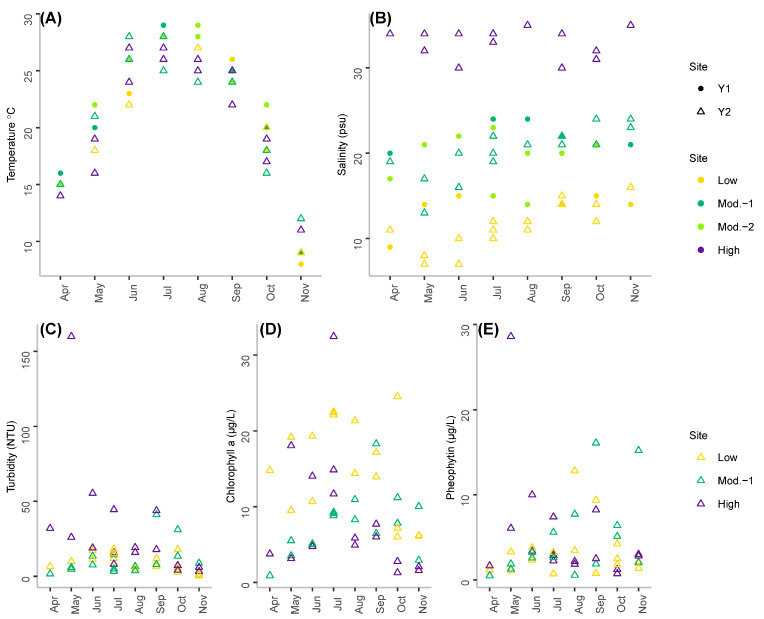
Environmental parameters measured during the study. (**A**) water temperature and (**B**) salinity measured at each site during both studied years. (**C**) turbidity, (**D**) chlorophyll *a* and (**E**) pheophytin measured in Year 2 of the study.

Water temperatures measured over the study period were similar across sites over the two sampling years (Figure 2A). The first sampling time points in April were associated with temperatures of approximately 15 °C, before increasing to values > 20 °C from June through September with the maximum temperature of 29 °C recorded in August. 

The lowest temperatures (15 °C down to 8 °C) were recorded in November.

As expected, the study sites encompassed a wide range of salinities (Figure 2B). The low salinity site was associated with salinities < 15 psu, while the high salinity site was associated with salinities > 32 psu. Salinities at the two moderate salinity sites ranged from 16 to 22 psu.

During the second study year when turbidity chlorophyll *a* and pheophytin were measured, the range of turbidity values at the three studied sites ranged from 0.5 to 160.0 NTU. The particularly high value of 160.0 NTU was observed only once at our high salinity site and all other values were <60.0 NTU (Figure 2C). Chlorophyll *a* concentrations ranged from 0.9 to 32.5 µg/L, with higher values more often recorded at our low salinity site (Figure 2D). Concentrations of pheophytin ranged from 0.5 to 28.6 µg/L with median values of 2.7 to 2.9 µg/L across the three studied sites (Figure 2E).

### 3.2. Dynamics of Total and Pathogenic Vibrio vulnificus

In both oyster and water samples, percent detection of *vvhA*+ was lower at the high salinity site (63% in water, 66% in oysters) compared to the other sites (>75%, see Table S2). Similarly, based on the analysis of all Year 2 samples, detection of *pilF*+ strains was lower at the high salinity site (25% in water, 28% in oysters) compared to the other sites (>75%).

**Figure 3:**
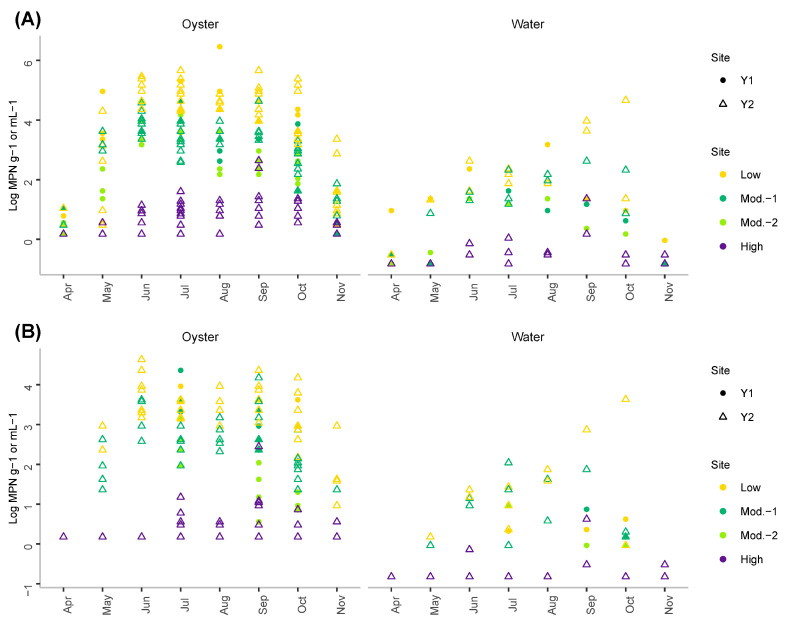
Concentrations of (**A**) total (*vvhA*) and (**B**) pathogenic (*pilF*) *V. vulnificus* measured during the study in oyster (left panel) and water samples (right panel).

Measured concentrations of *vvhA*+ (Figure 3A) and *pilF*+ (Figure 3B) in both oyster and water samples were also lower at the high salinity site compared to the other sites. At the other sites, the concentrations of both *vvhA*+ and *pilF*+ strains followed a seasonal cycle with the lowest concentrations observed in April and November. In oysters, concentrations reached up to 2.9 × 10^7^ MPN/g and 4.3 × 10^4^ MPN/g for *vvhA*+ and *pilF+*, respectively. In water samples, concentrations reached up to 4.6 × 10^4^ MPN/mL and 4.3 × 10^3^ MPN/mL for *vvhA*+ and *pilF+*, respectively. These values were all measured at the low salinity site reflecting a tendency for this site to be associated with higher concentrations of *V. vulnificus* compared to the moderate and high salinity sites.

### 3.3. Dynamics of Total and Pathogenic Vibrio parahaemolyticus

Considering each site and study year separately, detection of the *tlh* gene associated with total *V. parahaemolyticus* occurred in a majority of the collected samples. Percent detection for this species at each site ranged from 86 to 98% and from 75 to 100% in oyster and water samples, respectively (Table S2). Percent detection of *tdh*+ or *trh*+ in oyster samples ranged from 25 to 67% and from no detection to 54%, respectively. In water samples, the percent of detection was lower than in oysters with a maximum of 25% for *tdh*+ and 38% for *trh*+.

**Figure 4:**
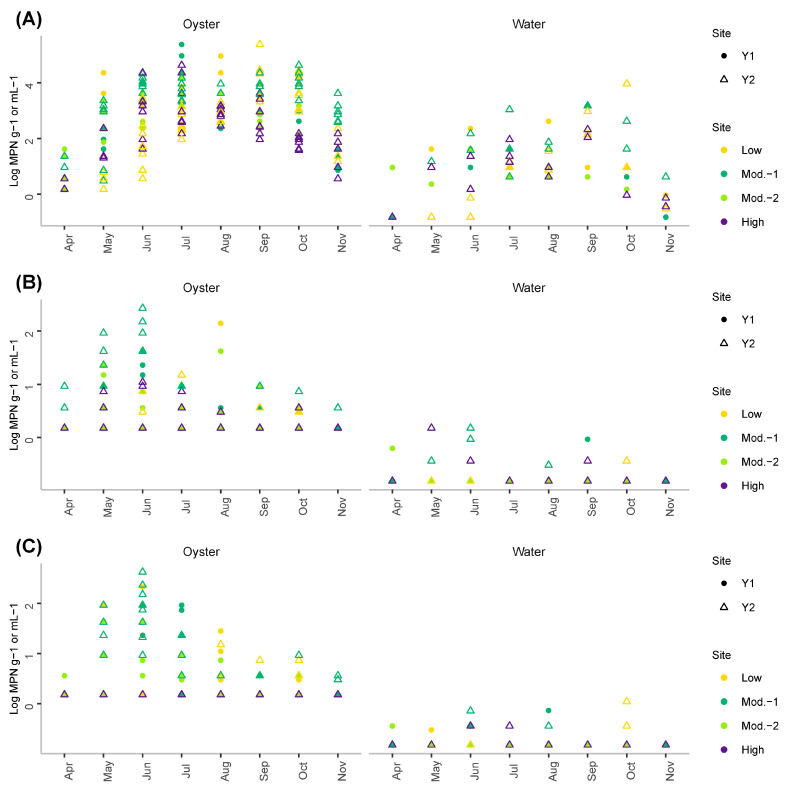
Concentrations of (**A**) total *V. parahaemolyticus* (*tlh*), (**B**) pathogenic *V. parahaemolyticus tdh*+ and (**C**) pathogenic *V. parahaemolyticus trh*+ measured in oysters (**left panel**) and water samples (**right panel**).

Concentrations of total *V. parahaemolyticus* in oysters and in water followed a seasonal cycle with lower values observed during the coldest months of the study, i.e., April and November (Figure 4A). Concentrations as high as 1.10 × 10^7^ MPN/mL and 1.9 × 10^3^ MPN/mL were recorded for this species in oyster and water samples, respectively. In oysters, *tdh*+ (Figure 4B) and *trh*+ strains (Figure 4C) presented different dynamics compared to total *V. parahaemolyticus* with higher concentrations detected from late spring to early summer. A similar trend was not evident in the water likely due to the small range of concentrations measured. *tdh*+ concentrations were as high as 270 MPN/g and 1.5 MPN/mL in oyster and water samples, respectively. Similarly, *trh*+ concentrations were as high as 420 MPN/g and 1.1 MPN/mL in oyster and water samples, respectively.

### 3.4. Associations between Total and Pathogenic Strains

**Figure 5:**
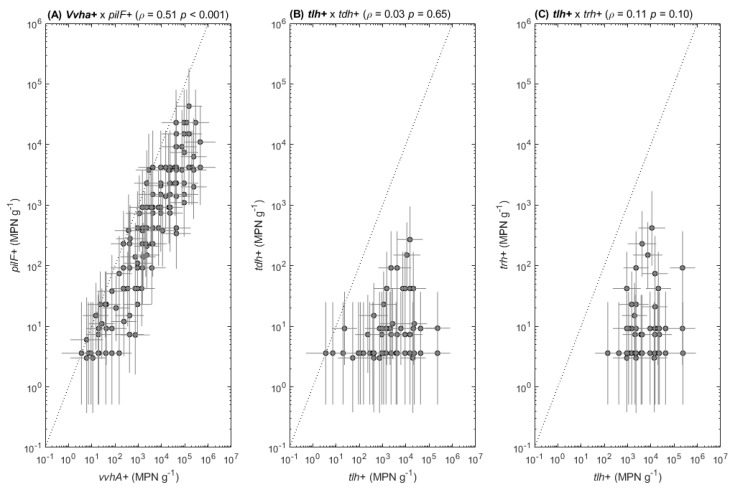
Pearson’s correlations between (**A**) concentrations of total (*vvhA*+) and pathogenic (*pilF*+) *V. vulnificus*, (**B**) concentrations of total (*tlh*+) and pathogenic *V. parahaemolyticus tdh*+, and (**C**) concentrations of total (*tlh*+) and pathogenic *V. parahaemolyticus trh*+ measured during both years of the study in oyster samples. The dashed lines illustrate a 1:1 identity line.

The potential association between measured concentrations of total and pathogenic strains in oyster samples was examined. A significant association (*ρ* = 0.51, *p* < 0.001) was observed between total *V. vulnificus* and *pilF*+ strains (Figure 5A). In contrast, the associations between total *V. parahaemolyticus* and *tdh*+ strains (*ρ* = 0.03, *p* = 0.65) or *trh*+ strains (*ρ* = 0.11, *p* = 0.10) were not significant (Figure 5B,C).

### 3.5. SEM Results

#### 3.5.1. Total (*vvha*+) and *pilF*+ *Vibrio vulnificus*

**Figure 6:**
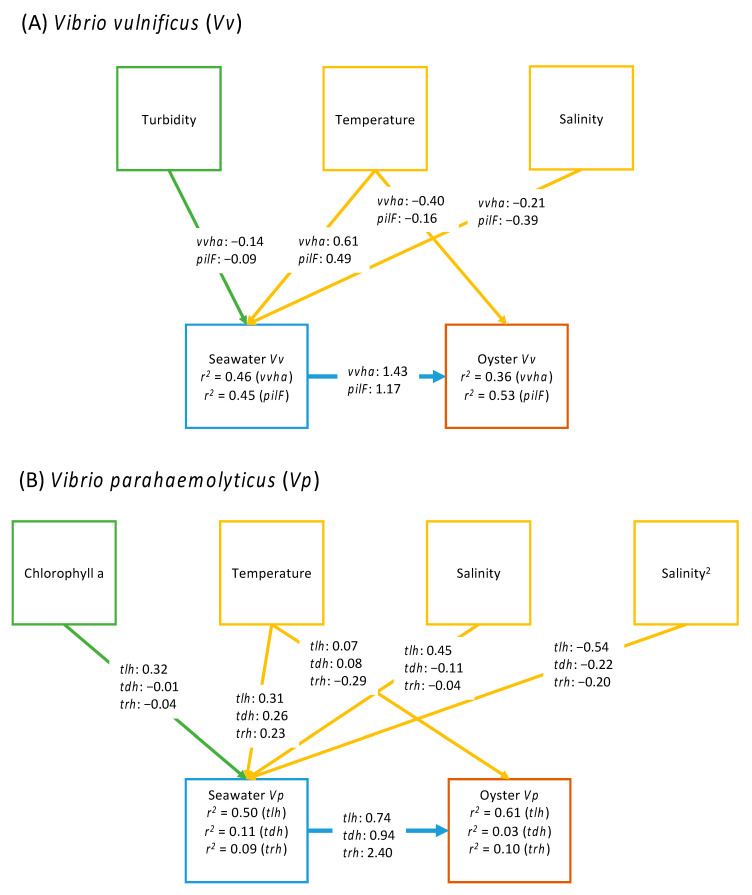
Results for the final selected models for each of the *Vibrio vulnificus* gene targets ((**A**): *vvha*: χ^2^ = 0.23, df = 1, *p* = 0.63; *pilF*: χ^2^ = 3.80, df = 1, *p* = 0.05) and *V. parahaemolyticus* gene targets ((**B**): *tlh*: χ^2^ = 3.79, df = 2, *p* = 0.05; *tdh*: χ^2^ = 3.25, df = 2, *p* = 0.20; *trh*: χ^2^ = 3.63, df = 2, *p* = 0.16). Boxes represent observed variables and path coefficients are standardized values (standardized by the standard deviation of the variables) for each gene target. Proportional variance explained by the models for all dependent variables (*Vibrio* spp. concentrations in seawater and oysters) are shown with each of these variables. Note the linear relationship between salinity and seawater *V. vulnificus* concentrations (*vvhA* and *pilF*) but convex relationship (specifying the salinity^2^ term) between salinity and seawater *V. parahaemolyticus* (*tlh*, *tdh*, and *trh*).

We found the most evidence in our collected data for iterations of Model A that included turbidity over chlorophyll *a* and pheophytin, to describe associations between primary productivity and seawater concentrations of *vvha*+ and *pilF*+ (Table S3). Similarly, we found stronger evidence for iterations of Model B that included turbidity over the other primary productivity proxies, again for *vvha+* and *pilF+*. We found consistently more evidence for iterations of Model B over Model A, where Model B iterations that included turbidity as a covariate yielded chi-square values of 0.23 (df = 1, *p* = 0.63) and 3.80 (df = 1, *p* = 0.06) for respective *vvha*+ and *pilF*+ concentrations in seawater and oysters. Note that *p* values greater than 0.05 indicate no major discrepancies between the model and data. From these results, we accepted Model B with turbidity as a covariate for both *vvha*+ and *pilF*+. The standardized path coefficients and variance explained in the measured *vvha*+ and *pilF*+ concentrations in seawater and oysters are shown in Figure 6. Interestingly, for both *vvha*+ and *pilF*+ water temperatures were positively associated with seawater concentrations, but negatively associated with concentrations in oysters once we controlled for the effect of seawater *Vibrio* concentrations on concentrations measured in oysters (Figure 6). Seawater *vvha*+ and *pilF*+ concentrations were a stronger predictor of these targets in oysters compared to temperature alone. Overall, these model results reveal that *vvha*+ and *pilF*+ *V. vulnificus* concentrations in oysters follow their concentrations in the surrounding seawater.

#### 3.5.2. Total (*tlh*+), *tdh*+, and *trh*+ *Vibrio parahaemolyticus*

Similar to *V. vulnificus*, we found consistently more evidence for iterations of Model B over Model A with total (*tlh+*)*, tdh+,* and *trh*+ *V. parahaemolyticus* concentrations as outcomes (Table S3). We found equivocal results among the three proxy variables for primary productivity, where we found more evidence in our collected data for iterations of Model B that included chlorophyll *a* for *tlh*+ and *trh*+, but more evidence for turbidity with *tdh*+ (Table S3). Model B iterations that included chlorophyll *a* as a covariate yielded chi-square values of 3.79 (df = 2, *p* = 0.05) and 3.63 (df = 2, *p* = 0.16) for respective *tlh*+ and *trh*+ *V. parahaemolyticus* concentrations in seawater and oysters, while the Model B iteration for *tdh*+ *V. parahaemolyticus* concentrations in seawater and oysters which included turbidity as a covariate yielded a chi-square value of 5.62 (df = 2, *p* = 0.06). Similar to *V. vulnificus*, water temperatures were positively associated with seawater *tdh*+ *V. parahaemolyticus* concentrations, but negatively associated with these concentrations in oysters (Figure 6). For *tlh*+ and *trh*+, water temperatures were positively associated with concentrations measured in water, while the association between water temperature and concentrations of these targets in oysters was more neutral (Figure 6). Again, similar to *V. vulnificus*, seawater *V. parahaemolyticus* concentrations were a stronger predictor of *V. parahaemolyticus* in oysters, revealing that *tlh*+, *tdh*+, and *trh*+ *V. parahaemolyticus* concentrations in oysters follow their concentrations in the surrounding seawater.

## 4. Discussion

The identification of the factors influencing levels of human-pathogenic *Vibrio* spp. in oysters is critical for developing accurate predictive models and improving risk management with regard to these pathogens. Our SEM results suggest that in some locations, models based on data associated with overlying seawater samples, may have value for predicting the dynamics of these pathogens in oysters. Within our studied oyster grow-out sites located in the lower Chesapeake Bay region, concentrations of all five gene targets analyzed (*vvhA*+, *pilF*+, *tlh*+, *tdh*+ and *trh*+) measured in oysters responded positively to concentrations of these targets measured in the seawater. In addition, seawater concentrations were a better predictor of oyster concentrations than temperature. This suggests that the overall dynamics of these *Vibrio* species or strains in oysters in terms of seasonality or in terms of site-specific responses followed the dynamics observed in surrounding waters, and/or that concentrations in seawater and oysters are driven by a set of similar factors.

Taken together our study and previous studies suggest, however, that our observations cannot be generalized to any oyster grow-out site or region. Both Zimmerman et al. [9] who focused on total and pathogenic *V. parahaemolyticus* at sites located in the Gulf Coast region, and Froelich et al. [7] who focused on total *V. vulnificus* and total *V. parahaemolyticus* at sites located in North Carolina, reported similar associations between seawater and oyster *Vibrio* concentrations as our study. However, the extensive study conducted by Nilsson et al. [8] within the Pacific Northwest region clearly demonstrated an absence of association between concentrations of total and pathogenic *V. parahaemolyticus* measured in seawater and oysters. This lead Nilsson et al. [8] to conclude that water sample analysis to assess risk associated with oyster consumption was inadequate in the studied region. In that region as well as in some of our sites, oysters were grown in the intertidal area, so they were exposed to air at low tide and we hypothesize that tidal range may be one of the key factors explaining the discrepancies between our results and those from the Pacific Northwest. As previously shown, at low tide oysters grown intertidally can be associated with increased *Vibrio* species levels as a result of exposure to warm air temperature [43,44,45]. The extent to which *Vibrio* species levels increase in oysters grown in the intertidal is primarily influenced by ambient air temperature, and by tidal range driving the duration of the air exposure. In two of our study sites as in the study conducted by Zimmerman et al. [9], oysters remained submerged preventing temperature increases associated with exposure to air at low tide. In our two other sites, oysters could be exposed to air a low tide, however, within the Chesapeake Bay region, the mean tidal range is <1 m, which contrasts with the tidal range of >3 m observed within the region studied by Nilsson et al. [8]. We hypothesize that the longer exposure to air of oysters grown under elevated tidal range may contribute to the lack of association between levels of pathogenic *V. parahaemolyticus* observed in oysters with levels of these strains in the water. Conversely, in regions located in low tidal range such as the mid-Atlantic and the Gulf coast, associations between human pathogenic *Vibrio* species in oysters and their surrounding seawater can be observed [7,9].

Results obtained through SEM models reiterated the influence of temperature and to a lesser extent of salinity, on human-pathogenic *Vibrio* spp. [6]. However, with regard to the concentrations of these bacteria in oysters, concentrations measured in water were a better predictor than these parameters. In water samples, measures of turbidity or chlorophyll *a* were also included as predictor variables for three (*vvhA*+, *pilF*+ and *tdh*+) or two of our gene targets (*tlh*+, *trh*+), respectively. SEM results suggested, however, that at our sites these parameters are weaker predictors of the concentrations of these bacteria compared to temperature, salinity and to seawater *Vibrio* concentrations. Although our results showed consistent associations between seawater *Vibrio* concentrations and *Vibrio* concentrations measured in oysters for all gene targets, these associations were not as strong for the pathogenic *V. parahaemolyticus* strains. This was due to the overall low concentrations of these targets in seawater and oysters, so improving our ability to detect low abundance of these strains in water samples, for example by increasing the volume inoculated in APW will be critical to better describe the dynamics of these strains in the water.

At our study sites, the abundance of potentially pathogenic *pilF*+ *V. vulnificus* strains measured in oysters paralleled that of total *V. vulnificus* in terms of temporal dynamics and distribution among sites. The influence of temperature and salinity patterns are well-known for total *V. vulnificus* [11,46,47,48,49], but very little is known with regard to the dynamics of potentially pathogenic *V. vulnificus* strains. One of the reasons is the lack of consensus regarding the appropriate virulence marker for these strains. In this study, we assessed the concentrations of pathogenic *V. vulnificus* through the detection of *pilF*+ strains in MPN enrichments using qPCR. To our knowledge, this was the first time the qPCR assay designed by Baker-Austin et al. [27] was applied to such samples. Previous studies focusing on the characterization of pathogenic *V. vulnificus* have explored additional virulence markers based on 16S rRNA polymorphism [29,30,31], or based on the virulence-coded gene (*vcg*) [32,33,34]. Studies using *vcg* as a marker have suggested that the dynamics of pathogenic (or clinical) *V. vulnificus* may be influenced not only by the isolate source but also by site-specific conditions [29,50,51,52]. Interestingly, in spite of differences in terms of marker (*pilF* versus *vcg*), and sample processing methods (MPN approach versus single isolates), a positive association between total and pathogenic *V. vulnificus* was observed in both our study and Williams et al. [51]. Based on these observations, we may hypothesize that elevated levels of total *V. vulnificus* are accompanied by elevated levels of pathogenic *V. vulnificus*, thereby enabling measurements of total *V. vulnificus* as a measure of risk for humans. We acknowledge nevertheless that caution should be used in interpreting our results and in inferring an accurate measure of pathogenicity based on *pilF*+ concentrations. Indeed, while Dickerson et al. [14] showed that the efficacy of the *pilF* assay [27] to distinguish clinical and environmental isolates was comparable to 16S rRNA polymorphisms [29,30,31] or *vcg* [32,33,34], these authors also acknowledged that none of the above virulence markers definitively distinguish all clinical from environmental isolates. In fact, the proportion of clinical isolates correctly identified using each of these assays ranged from 74.1 to 79.2% [14]. This continues to underscore the need to find more accurate virulence markers for *V. vulnificus* [2,14,28,53] but it also shows that *pilF*, although imperfect, provides some information relative to the pathogenicity potential of the strains carrying this gene.

In contrast to what we observed with total and pathogenic *pilF*+ *V. vulnificus*, total and pathogenic *V. parahaemolyticus* differed in terms of their dynamics, notably in terms of their seasonal dynamics. Within our studied sites, pathogenic *V. parahaemolyticus* concentrations peaked in the water and in oysters in June and then declined, while concentrations of total *V. parahaemolyticus* remained elevated during July, August and September in most samples and started decreasing in the fall. Other studies conducted within the lower Chesapeake Bay region and relying on samples collected from 2014 to 2019 have highlighted similar dynamics of these bacteria in oysters [43,54,55]. These data suggest that the trends observed in this study are stable over-time or at least under this time scale. Differential in situ dynamics between total and pathogenic *V. parahaemolyticus* strains has been observed in other studies [9,51,55,56,57]. While the actual biotic and/or abiotic factors driving these different in situ responses remain to be identified, results of in vivo studies exploring the role of genes associated with pathogenic strains suggest that the role of biotic interactions should be further investigated [58,59].

Our data suggest that concentrations of human-pathogenic *Vibrio* spp. measured in water have some value for risk assessment in low tidal range grow-out regions. When feasible, we nevertheless acknowledge that *Vibrio* spp. concentrations in oysters remain the optimal measure of risk with regard to oyster consumption [60]. One of the key reasons lies in the observed intrapopulation variability in levels of these bacteria in oysters. Previous studies focusing on individual oysters have shown that levels of human-pathogenic *Vibrio* spp. can vary over several orders of magnitude, suggesting that the influence of the oyster itself cannot be discounted [54,55,56,61,62,63]. Additionally, higher concentrations of these bacteria measured in oysters compared to water samples facilitate detection in oyster samples, especially with regard to *tdh*+ and *trh*+ strains. As observed previously, pathogenic *V. parahaemolyticus* measurements in water were associated with lower detection percentage compared to oyster samples [49,64] hindering characterization of their dynamics and development of predictive models.

## 5. Conclusions

Within oyster grow-out areas of the lower Chesapeake Bay region, we observed a positive association between concentrations of human-pathogenic *Vibrio* species measured in seawater and the concentrations of these bacteria measured in oysters. Based on results obtained in previous studies, we hypothesize that this association may be influenced by the tidal range. More specifically, we hypothesize that concentrations of these bacteria in seawater and oysters are coupled under low tidal range conditions, but become decoupled in regions exposed to high tidal range conditions. We suggest that under low tidal range conditions, monitoring seawater and water-based predictive models have some value for assessing risks associated with oyster consumption. At smaller spatial scales, other factors not explored in our study such as water depth and hydrodynamics may also influence the degree of associations occurring between seawater and oyster bacterial concentrations and these factors may warrant further investigation. Our results suggest that the occurrence of potentially pathogenic *V. vulnificus* measured through detection of the *pilF* gene and total *V. vulnificus* paralleled each other. Our study also underscores the unique dynamics of pathogenic *V. parahaemolyticus* compared to total *V. parahaemolyticus* and the need to better understand the ecology of these pathogenic strains.

## Data Availability

The original contributions generated for the study are included in the article; further inquiries can be directed to the corresponding author.

## Supplementary Materials

The following supporting information can be downloaded at: https://www.mdpi.com/article/10.3390/foods11244065/s1, Table S1. Quantitative PCR primers and probes used in this study; Table S2. Percentage of positive samples for each gene targeted in the study; Table S3: Model fit statistics from all fitted models for all *Vibrio* targets. Shown for each model is the chi-squared test statistic (χ2) and model degrees of freedom (df) with associated P value, Akaike Information Criterion (AIC), difference in Akaike Information Criteria between each model and the best fit model within each group (dAIC; we defined each group as the full suite of iterations of Model A and Model B testing each Chlorophyl *a*, Pheophytin, and Turbidity as covariates) and the coefficients of determination for the two outcomes, *Vibrio* concentrations measured in seawater (r2 water) and oyster tissue homogenates (r2 oyster).
