# Supplementary material for: Vibrio vulnificus and Vibrio parahaemolyticus in Oysters under Low Tidal Range Conditions: Is Seawater Analysis Useful for Risk Assessment?
Source: Foods. 2022 Dec 16;11(24):4065. doi: 10.3390/foods11244065 (PMC9778087; doi:10.3390/foods11244065)
Supplement: Supplementary file 1 [file foods-11-04065-s001.zip › foods-2014928-supplementary.pdf]

Supplementary Materials

Vibrio vulnificus and *Vibrio parahaemolyticus* in oysters under low tidal range conditions: is seawater analysis useful for risk assessment?

Corinne Audemard[1], Tal Ben-Horin[2], Howard I. Kator[1] and Kimberly S. Reece[1]

[1] Virginia Institute of Marine Science, William & Mary, P.O. Box 1346, Gloucester Point, Virginia, USA

[2] College of Veterinary Medicine, North Carolina State University, Morehead City, NC, USA

Table S1: Quantitative PCR primers and probes used in this study.

Primer or Probe	Sequence (5’ to 3’)	Modifications	Source
tlh forward	ACTCAACACAAGAAGAGAT CGACAA		[13]
tlh reverse	GATGAGCGGTTGATGT CCAA		
tlh probe	CGCTCGCGTTCACGAAA CCGT	5’ TxRED to 3’ BHQ2	
tdh forward	TCCCTTTTCCTGCCCCC		
tdh reverse	CGCTGCCATTGTATAGTCTT TATC		
tdh probe	TGACATCCTACATGAC TGTG	5’ FAM to 3’ MGBNFQ	
trh forward	TTGCTTTCAGTTTGCTATT GGCT		
trh reverse	TGTTTACCGTCATATAGGC GCTT		
trh probe	AGAAATACAACAATCAAAA CTGA	5’ NED to 3’ MGBNFQ	
IAC forward	GACATCGATATGGGTGCCG		
IAC reverse	CGAGACGATGCAGCCATTC		
IAC probe	TCTCATGCGTCTCCCTGGTGAATGTG	5’ Cy5 to 3’ BHQ2	
vvhA forward	TGTTTATGGTGAGAACGGTGACA		[25]
vvhA reverse	TTCTTTATCTAGGCCCCAAACTTG		
vvhA probe	CCGTTAACCGAACCACCCGCAA	5’ TAMRA to 3’ FAM	
pilF forward	GATTGACTACGAYCCACACCG		[27]
pilF reverse	GRCGCGCTTGGGTGTAG		
pilF probe	TGCTCAACCTCGCTAAGTTGGAAATCGATAC	5’ TAMRA to 3’ FAM	

Table S2: Percentage of positive samples for each gene targeted in the study

	Y1			Y2		
	Low	Mod.-1	Mod.-2	Low	Mod.-1	High
	% of detection of each target (number of positives/total number of analyzed samples)					
vvhA+-oyster	88 (28/32)	81 (32/26)	89 (25/28)	92 (59/64)	84 (54/64)	66 (42/64)
vvhA+-water	100 (8/8)	75 (6/8)	86 (6/7)	81 (13/16)	81 (13/16)	63 (10/16)
pilF+ -oyster	100 (10/10)	100 (8/8)	100 (12/12)	83 (53/64)	81 (52/64)	28 (18/64)
pilF+ -water	100 (3/3)	100 (3/3)	100 (3/3)	75 (12/16)	75 (12/16)	25 (4/16)
tlh+-oyster	91 (29/32)	91 (29/32)	96 (27/28)	86 (55/64)	98 (63/64)	66 (42/64)
tlh+-water	88 (7/8)	75 (6/8)	100 (7/7)	75 (12/16)	88 (14/16)	88 (14/16)
tdh+-oyster	25 (8/32)	34 (11/32)	39 (11/28)	28 (18/64)	67 (43/64)	25 (16/64)
tdh+-water	0 (0/8)	13 (1/8)	14 (1/7)	6 (1/16)	25 (4/16)	25 (4/16)
trh+-oyster	44 (14/32)	34 (11/32)	54 (15/28)	17 (11/64)	53 (34/64)	0 (0/64)
trh+-water	13 (1/13)	38 (3/8)	14 (1/7)	13 (2/16)	25 (4/16)	13 (2/16)

Table S3: Model fit statistics from all fitted models for all *Vibrio* targets. Shown for each model is the chi-squared test statistic (χ^2) and model degrees of freedom (df) with associated P value, Akaike Information Criterion (AIC), difference in Akaike Information Criteria between each model and the best fit model within each group (dAIC; we defined each group as the full suite of iterations of Model A and Model B testing each Chlorophyl a, Pheophytin, and Turbidity as covariates) and the coefficients of determination for the two outcomes, *Vibrio* concentrations measured in seawater (r^2 water) and oyster tissue homogenates (r^2 oyster).

Target	Model	Model covariates	χ^2	df	P	AIC	dAIC	r^2 water	r^2 oyster
vvhA	A	Oysters ~ 1 + Temp. + Sal.							
		Water ~ 1 + Temp. + Sal. + Chlo.	0.632	1	0.632	2822.743	11.262	0.437	0.347
		Oysters ~ 1 + Temp. + Sal.							
		Water ~ 1 + Temp. + Sal. + Pheo.	3.389	1	0.066	2823.371	11.89	0.437	0.347
		Oysters ~ 1 + Temp. + Sal.							
		Water ~ 1 + Temp. + Sal. + Turb.	10.174	1	0.001	2821.423	9.942	0.462	0.347
vvhA	B	Oysters ~ 1 + Water + Temp.							
		Water ~ 1 + Temp. + Sal. + Chlo.	1.457	1	0.227	2823.567	12.086	0.451	0.362
		Oysters ~ 1 + Water + Temp.							
		Water ~ 1 + Temp. + Sal. + Pheo.	0.009	1	0.922	2819.991	8.51	0.456	0.325
		Oysters ~ 1 + Water + Temp.							
		Water ~ 1 + Temp. + Sal. + Turb.	0.233	1	0.63	2811.481	0	0.462	0.362
pilF	A	Oysters ~ 1 + Temp. + Sal.							
		Water ~ 1 + Temp. + Sal. + Chlo.	1.448	1	0.229	2854.011	3.218	0.443	0.425
		Oysters ~ 1 + Temp. + Sal.							
		Water ~ 1 + Temp. + Sal. + Pheo.	0.389	1	0.533	2859.102	8.309	0.451	0.425
		Oysters ~ 1 + Temp. + Sal.							
		Water ~ 1 + Temp. + Sal. + Turb.	5.565	1	0.018	2853.793	3	0.45	0.425
pilF	B	Oysters ~ 1 + Water + Temp.							
		Water ~ 1 + Temp. + Sal. + Chlo.	0.356	1	0.551	2852.92	2.127	0.444	0.558
		Oysters ~ 1 + Water + Temp.							
		Water ~ 1 + Temp. + Sal. + Pheo.	5.269	1	0.022	2853.983	3.19	0.445	0.567
		Oysters ~ 1 + Water + Temp.							
		Water ~ 1 + Temp. + Sal. + Turb.	3.803	1	0.056	2850.793	0	0.45	0.533
tlh	A	Oysters ~ 1 + Temp. + Sal.							
		Water ~ 1 + Temp. + Sal. + Chlo.	2.539	1	0.111	2234.208	4.167	0.438	0.338
		Oysters ~ 1 + Temp. + Sal.							
		Water ~ 1 + Temp. + Sal. + Pheo.	0.33	1	0.565	2246.336	16.295	0.444	0.338
		Oysters ~ 1 + Temp. + Sal.							
		Water ~ 1 + Temp. + Sal. + Turb.	0.369	1	0.543	2248.978	18.937	0.438	0.338

tlh	B	Oysters ~ 1 + Water + Temp.<br>Water ~ 1 + Temp. + Sal. + Chlo.	3.789	2	0.052	2230.041	0	0.502	0.615
		Oysters ~ 1 + Water + Temp.<br>Water ~ 1 + Temp. + Sal. + Pheo.	5.477	2	0.065	2249.483	19.442	0.446	0.611
		Oysters ~ 1 + Water + Temp.<br>Water ~ 1 + Temp. + Sal. + Turb.	5.078	2	0.079	2251.686	21.645	0.438	0.61
tdh	A	Oysters ~ 1 + Temp. + Sal.<br>Water ~ 1 + Temp. + Sal. + Chlo.	0.694	1	0.405	2882.407	26.216	0.119	0.145
		Oysters ~ 1 + Temp. + Sal.<br>Water ~ 1 + Temp. + Sal. + Pheo.	0.007	1	0.934	2876.06	19.869	0.146	0.145
		Oysters ~ 1 + Temp. + Sal.<br>Water ~ 1 + Temp. + Sal. + Turb.	1.419	1	0.234	2853.988	-2.203	0.237	0.145
tdh	B	Oysters ~ 1 + Water + Temp.<br>Water ~ 1 + Temp. + Sal. + Chlo.	3.249	2	0.197	2882.961	26.77	0.112	0.031
		Oysters ~ 1 + Water + Temp.<br>Water ~ 1 + Temp. + Sal. + Pheo.	5.163	2	0.076	2879.217	23.026	0.143	0.027
		Oysters ~ 1 + Water + Temp.<br>Water ~ 1 + Temp. + Sal. + Turb.	5.621	2	0.06	2856.191	0	0.246	0.136
trh	A	Oysters ~ 1 + Temp. + Sal.<br>Water ~ 1 + Temp. + Sal. + Chlo.	2.431	1	0.119	2821.771	0.806	0.105	0.304
		Oysters ~ 1 + Temp. + Sal.<br>Water ~ 1 + Temp. + Sal. + Pheo.	0.555	1	0.456	2821.505	0.54	0.106	0.304
		Oysters ~ 1 + Temp. + Sal.<br>Water ~ 1 + Temp. + Sal. + Turb.	0.629	1	0.428	2821.345	0.38	0.107	0.304
trh	B	Oysters ~ 1 + Water + Temp.<br>Water ~ 1 + Temp. + Sal. + Chlo.	3.625	2	0.163	2820.965	0	0.089	0.096
		Oysters ~ 1 + Water + Temp.<br>Water ~ 1 + Temp. + Sal. + Pheo.	3.061	2	0.216	2822.012	1.047	0.094	0.093
		Oysters ~ 1 + Water + Temp.<br>Water ~ 1 + Temp. + Sal. + Turb.	3.154	2	0.207	2821.87	0.905	0.091	0.095
